# Natural Self-Ligand Gamma Delta T Cell Receptors (γδTCRs) Insight: The Potential of Induced IgG

**DOI:** 10.3390/vaccines8030436

**Published:** 2020-08-04

**Authors:** Thamires Rodrigues de Sousa, Jefferson Russo Victor

**Affiliations:** 1 Laboratory of Medical Investigation LIM-56, Division of Clinical Dermatology, Medical School, University of Sao Paulo, Sao Paulo 05403-000, Brazil; sousarthamires@gmail.com; 2 Division of Environmental Health, FMU, Laureate International Universities, Sao Paulo 04505-002, Brazil

**Keywords:** gamma delta T, gamma delta TCR, gamma delta TCR ligands, IgG

## Abstract

A γδ T cell acquires functional properties in response to the gamma delta T cell receptor γδTCR signal strength during its development in the thymus. The elucidation of the potential ligands of γδ T cell receptors are of extreme importance; however, they are still not understood. Here we revise the actual state of the art of candidates to exert the function of γδTCR ligands, and propose a theoretical contribution about new potential ligands of γδTCRs, based on biological and hypothetical pieces of evidence in the literature. In conclusion, we hypothetically suggest a possible role of induced antibodies according to the individual’s immune status, mainly of the IgG subclass, acting as γδTCR ligands. Considering that IgG production is involved in some essential immunotherapy protocols, and almost all vaccination protocols, our discussion opens a new and broad field to further exploration.

## 1. γδ T Cells

Matured in the mouse and human thymus, γδ T cells are characterized by expressing T cell receptors TCRs composed of γ and δ chains, and represent the major resident T cell population [1]. In response to activation, these cells can produce several cytokines and chemokines with several modulatory and regulatory functions on innate and adaptive immunity [2,3,4].

This ability to transition between innate and adaptive immunity is a typical feature of this cell population, given by non-major histocompatibility complex non-MCH restricted antigenic specificity [5], as is the capability of initiating rapid immune responses to a large number of potential tissue stressors, exerting functions related to homeostasis and the host defense of epithelial barrier tissues, and the lymphoid stress-surveillance response [1,2].

Being one of the first generated T cells in the embryonary period, γδ T cells quickly spread to the peripheral tissues but are not found with a high frequency in the lymphoid tissues and blood of adult individuals. On the other hand, γδ T cells are detected at a higher rate in mucosal and epithelial tissues [6,7]. In the mucosal environment, these cells are activated in response to stress, including environmental challenges, infections, and malignant transformation from the surrounding tissues, and they perform many functions depending upon the location and type of stress that has occurred, in other words, γδ T cells act as sentinels [8].

In humans, about 5–40% of intraepithelial lymphocytes (IELs) in the paracellular space are γδ T cells [9]. The γδTCR repertoire in the human intestine undergoes many changes during the development from fetus to adult. Thus, γδ T cells may mediate different functions at each stage of maturation. Since the γδTCR repertoire is oligoclonal and stable over time, γδ T cells may recognize a limited array of antigens that are highly conserved among different bacterial strains, mostly intracellular microbial pathogens (e.g., *Listeria*, *Mycobacterium*, *Plasmodia*, *Toxoplasma*). Additionally, γδ IEL have been shown to produce cytokines and growth factors, and to influence epithelial cell proliferation and differentiation, as well as the mucosal development of immunoglobulin A (IgA) secreting B cells [10].

About 15–25% of T cells in the liver express the γδ TCR and thus exert essential functions in the homeostasis and diseases of this organ. It is also well known as a site of the extrathymic development of γδ T cells during the human fetal period [11]. These cells have reportedly been shown to have their subsets altered during the progression of liver diseases, as they are probably of great importance in determining the fate of the inflammatory process in this organ [12]. A great example is γδ T cells contributing to hepatocyte apoptosis via Fas ligands, resulting in the engagement and recruitment of cytotoxic T cells in cases of hepatofibrogenesis, thereby limiting liver fibrosis [13].

Although γδ T cell subsets can be defined by the expression of the γ and δ chains, two significant γδ T cell subsets are defined by their capacity to produce IL-17 or IFN-γ in mice. These subsets are not established in humans [14]. In this regard, a study has revealed that, in mice, γδ T cells encountering the antigen in the thymus were neither required nor inhibitory for their development, but when triggered through the T cell receptor, naive γδ T cells produced IL-17, whereas ligand-experienced cells produced IFN-γ [15].

Previous studies demonstrated that IL-17-producing γδ T cells collaborate with an effective immune response against infections [16,17,18,19] and with the pathogenesis of autoimmunity [20,21]. These cells exert nonredundant immunological functions [22]. Otherwise, IFN-γ-producing γδ T cells are related to the development of murine fulminant viral hepatitis infection [23] and exert protective/regulatory functions in tumor immunity [24,25].

Unlike murine models, γδ T cell subsets in humans are generally defined based on the Vδ TCR chain, narrowing down into two major populations: Vδ2+ and Vδ2− γδ T cells. Vδ2+ T cells are developed mainly in the fetal liver and thymus [26,27], and are the predominant γδ T cell population in the peripheral blood of adult humans [28]. They can also be recruited to inflamed tissues to help with pathogen clearance or to promote inflammation. On the other hand, the Vδ2− γδ T cell subset can be found in epithelial tissues, such as the skin [29] and intestines [9], and appears to form resident populations in the liver [30]. They mainly consist of Vδ1+ T cells, with fewer Vδ3+ and Vδ5+ T cells.

It is also important to highlight that in humans, IFN-γ-producing γδ T cells can mediate the innate resistance to *Escherichia coli*, limiting systemic immunopathology [31]. Besides that, TCR-γδ T cells have emerged as essential players in allogeneic hematopoietic cell transplantation (alloHCT) and immune cell therapy [32].

Since the early 1990s, the relationship between specificity and Vγ and Vδ gene usage was described [28] and, currently, human γδ T cells can be classified according to the expression of δ genes (δ1-3) and γ genes (γ2-5, γ8, γ9, and γ11) [33]. Murine γδTCR chain expression can be related to a tissue site (e.g., γ1, γ4, and γ6 in the lung), and this differential expression influences the antigen-specific recognition properties of tissue-infiltrated γδ T cells between each tissue and, consequently, influences their collaboration with the development of an adaptive immune response [3].

Besides, in the context of adaptive immune responses, it was demonstrated that γδ T cells can favor murine antibody production by B cells by secreting IL-4 and IL-10 [34].

The γδTCR signaling during the early stages of maturation in the thymus can influence the mature γδ T cells’ cytokine secretion profile [35]. With the use of a molecule that can act as a ligand of γδTCRs, such as the major histocompatibility complex (MHC) class Ib ligands (H2-T10/22), it was demonstrated that γδTCR interaction can play an essential role in shaping the TCR repertoire of γδ T cells [36].

These observations indicate that the elucidation of the potential ligands of γδTCRs and their relations with the induction of functional properties in γδ T cells is of extreme importance. However, this has been discussed by renowned scientists in the literature since the late 1980s [37,38], and even after some evolutionary analysis, the mechanisms involved in γδTCR-dependent γδ T cell activation are still not understood [33]. The search for the identification of γδTCR ligands was named “the quest to solve a 500-million-year-old mystery” [39].

Taking into consideration the modulatory effects mediated by γδ T cells in the mechanism of immune inflammation, here we briefly revise the state of the art and propose a theoretical contribution to generate some discussions about new potential ligands of γδTCRs. With this purpose, we collected biological and hypothetical pieces of evidence from the literature.

## 2. Antigen Recognition Mechanism

As is known, αβT cells recognize antigens as antigen fragments after being processed by antigen-presenting cells (APC), followed by the binding of these fragments to various sites on the major histocompatibility complex (MHC) molecule of the APC, and posterior presentation at the cell surface to the TCR, and then activation and proliferation of the T cells [40,41,42,43,44]. However, there are far fewer pieces of evidence showing the mechanisms involved in antigen recognition by γδ T cells. However, it is essential to remember that these two lineages of cells differ not just at a molecular level, they also have a different antigenic repertoire, even though specific antigens are capable of stimulating both populations.

In this context, two significant points allow us to tell the difference between these two populations within the process of antigen recognition. The first is that some γδ T cells do not seem to require the classical MHC for antigen recognition, since the majority of γδ T cell hybridomas do not require the MHC for activation [45]. Furthermore, studies from another group have gathered data on a murine TCR gamma delta clone (TgI4.4) capable of recognizing a herpes simplex virus type 1 (HSV-1) transmembrane glycoprotein in an MHC class I- and II-independent manner [46]. On the other hand, there are shreds of evidence that γδ T cells may recognize some antigens, like tetanus toxoid, in an MHC-restricted fashion [47].

Although some γδ T cells were able to recognize proteins without processing, such as mouse class II MHC molecule IE^k^, T10, T22, and herpes simplex virus glycoprotein I, others were unable to recognize peptides bound to these proteins [48,49,50,51]. In addition, these studies have demonstrated that some γδ T cell clones seem to recognize antigens without the need for intracellular antigen processing, which means this recognition process is made directly on the surface.

Taken together, these pieces of evidence allow us to suggest that γδ T cells can recognize unprocessed antigens at the cell surface, exempting APCs as a requirement for antigen recognition by those cells. However, APCs may exert other functions when they can be stimulated by the products of gamma delta T cell activation into producing cytokines and growth factors, collaborating with the enhancement of the ongoing immunological response.

## 3. Evidenced and Proposed γδTCR Ligands

As discussed above, γδTCR signaling can determine the activity of γδ T cells [14,52,53], therefore a precise functional understanding of γδ T cells depends on the elucidation of the molecules capable of triggering their activation via γδTCRs.

Reasoning from this fact, here we will not discuss all pieces of the evidence of the direct interaction of several ligands with γδTCRs [54,55,56,57,58], but mainly those that are likely to generate future mechanistic or biological approaches in vivo.

First and foremost, a very recent study was able to demonstrate that γδTCR ligands can be expressed by several cells, including both human tumor cell lines and individual human primary cells. This study could also illustrate the spectrum of ligand(s) expression for human synovial Vδ1 γδ T cells, as well as the physiology that regulates their expression, but it did not accurately identify the molecules that can act as γδTCR ligands in those cells [59].

In this context, it was shown that spatially distinct regions of γδTCRs, as demonstrated by the hypervariable region 4 (HV4) of Vγ4 chains, can determine the antigen responsiveness of γδ T cells, increasing the complexity of the understanding of γδTCR ligand recognition [60].

**Figure 1:**
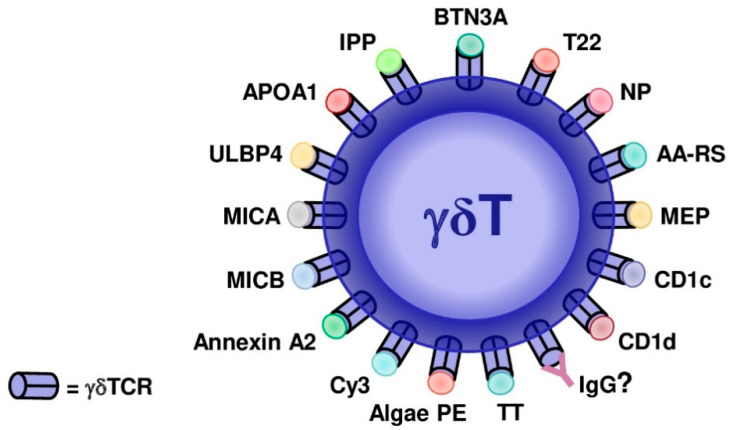
Evidenced and proposed γδTCR ligands in mice and humans. Butyrophilin-3 (BTN3A), nonclassical MHC molecule T22, 4-hydroxy-3-nitrophenylacetyl (NP), aminoacyl-tRNA synthetases (AA-RS), monoethyl phosphate (MEP), CD1c, CD1d, toxoid of *Clostridium tetani* (TT), algal phycoerythrin (PE), cyanine 3 (Cy3), annexin A2, MICB (MHC class I chain-related protein B), MICA (MHC class I chain-related protein A), ULBP4, APOA-1 (apolipoprotein A-1), isopentenyl pyrophosphate (IPP), and IgG are illustrated. ? = proposed non-evidenced ligand.

It has been shown that murine γδ T cells depend on the type of adjuvant used in immunization to shift the production of IL-17 [61]. On the other hand, it was described that human γδ T cell subsets can recognize both natural and synthetic phosphoantigens (pAgs) [62]. However, the mammalian molecule isopentenyl pyrophosphate (IPP) stimulates γδ T cells at a 10,000-fold higher concentration compared to phosphoantigens derived from bacteria. This last observation suggests that IPP cannot exert a physiological role as a γδTCR ligand [63]. Additionally, a report has indicated that pAgs do not interact directly with γδTCRs, in fact, the Vγ9Vδ2 T cell activation is due to the recognition of an allosteric change in the extracellular domain of a cell surface molecule, butyrophilin 3A1 [64]. The F1-ATPase molecule was proposed as a γδTCR ligand in a tumor recognition study [65], but the evidence for this is still incomplete. A detailed discussion about γδTCR ligands, summarized in Figure 1, and the knowledge generated in both species, will follow.

### 3.1. Algal Phycoerythrin (PE)

A noted B cell antigen, the algal protein phycoerythrin (PE), can act as a murine and human γδTCR antigen [66]. In this study, it was demonstrated that the recognition of algal PE by activated naive γδ T cells induces the production of IL-17 and yields the functionality to respond to cytokine signals, collaborating with the perpetuation of the immune response [66].

### 3.2. Annexin A2

More recently, using in vitro experiments in which tumor cells were exposed to various stress situations, it was observed that γδ T cells could recognize tumor cells. This direct recognition is mediated by a cell stress-related molecule, annexin A2. However, it seems that it occurs only with a specific subset of γδ T cells, the Vγ8Vδ3 TCR-expressing γδ T cells [67].

### 3.3. BTN3A (Butyrophilin-3)

A study using cell stress-related molecule phosphoagonist (PAg) up-regulation in tumor and mycobacteria-infected cells, demonstrated that a human γδ T cell could also recognize butyrophilin-3 (BTN3A) molecules. However, similar to annexin A2, it seems to occur only with a specific subset of γδ T cells, in this case, the Vγ9Vδ2 TCR-expressing γδ T cells [68]. Luckily, Vγ9Vδ2 T cells represent the major γδ T cell subset in human peripheral blood, with values ranging from 50% to 95% of γδ T cells, besides the fact that they stand out as being able to sense several infected and malignant cells [69].

### 3.4. T22

It was demonstrated that the non-classical MHC molecule, T22, could act as a ligand for γδTCRs in mice [70] and the MHC-like molecule, CD1, in mice and humans [71]. Performing a study that aimed to identify CD1d-sulfatide-specific T cells in healthy individuals, Bai L and collaborators surprisingly observed that the majority of fresh sulfatide-specific T cells belonged to the γδ lineage, and they mainly expressed Vδ1 chains in their TCRs [72]. This study provided the first demonstration of MHC-like-restricted, antigen-specific recognition by γδTCRs [72].

### 3.5. CD1c

The CD1c, a molecule expressed by human dendritic cells (DCs) and B cells when presenting antigens to T cells, can be recognized by γδTCRs when loaded with *Mycobacterium tuberculosis* phosphomycoketide [73]. This same study evidenced that the Vδ1 domain participates in recognition by γδTCRs and demonstrates that CD1c can complex with lipids, including lysophosphatidylcholine, sulfatide, and mannosyl-phosophomycoketide [73].

Although these molecules cannot mediate in vivo interaction with γδTCRs, shaping their maturation process, they may be especially crucial in the recognition of pathogens by peripheral γδ T cells. Otherwise, the definition of γδ T cells’ functions mainly occurs during theirmaturation in the thymus, suggesting that other ligands that can reach this organ are involved in the development of γδ T cell functions.

### 3.6. Haptens

Studies about γδ T cell hapten recognition emerged from the idea that these cells possibly recognize antigens similarly to B cells [48]. A study using immunized mice with Cy3-chicken gamma globulin (Cy3-CGG) in aluminum hydroxide demonstrated that haptens, such as cyanine 3 (Cy3) and 4-hydroxy-3-nitrophenylacetyl (NP), can be recognized directly by specific γδ TCRs, and are able to induce a T cell response [74]. Thus, these molecules are capable of up-regulating CD44^hi^ and CD62L^lo^, which is equal to an activated phenotype in γδ T cells.

### 3.7. Non-Peptides

The initial observation about non-peptide recognition was obtained with a non-peptide antigen derived from *M. tuberculosis* that could be recognized by murine and human γδ T cells [75]. From there, a subsequent study demonstrated that a variety of non-peptide molecules could stimulate human γδ T cells [76]. In the same study, it was shown that monoethyl phosphate (MEP), a synthetic alkyl phosphate, was able to activate these cells and stimulate their proliferation in vitro [76]. In both cases, all γδ T cells stimulated by these molecules expressed Vγ2/Vδ2 TCRs.

In a comparative approach, it was demonstrated that mycobacterial antigens recognized by γδ T cells were chemically and chromatographically similar to MEP, and both were able to expand primary Vy2/V82 T cells [76].

The human stress antigens MICA and MICB are MHC class I-related molecules originally described as ligands of NKG2D receptors expressed in NK cells. They have also been described as ligands of γδ T cells that express the variable region Vδ1, therefore acting as stress-induced self-antigens [77]. However, this correlation is limited to the intestinal epithelium because it corresponds to the environment where this subset of γδ T cells is prevalent, and where MICA and MICB are mainly expressed. The same study also showed that there is a correlation between these T cells and epithelial tumors, which, regardless of their type, express MICA/MICB. The reason behind this correlation is that the more MICA/MICB is expressed, the higher the number of γδ T cells found in the tissue [78]. In this regard, another study using soluble MICA tetramers as a binding reagent to demonstrate specific interactions with various Vδ1 γδ TCRs expressed on T cell lines lacking NKG2D, showed that MICA delivers both the TCR-dependent signal and the NKG2D-dependent costimulatory signal. This dual function may be useful to prevent inaccurate γδ T cell activation in cases of cross-reactivity with other cell surface compounds [79].

Another interesting molecule worth mentioning is ULBP4, which is also a ligand for NKG2D, along with MICA/MICB. In a study conducted by Kong’s group, ULBP4 not only can play a role as a ligand for TCRγδ, binding directly to it, but can also trigger the expansion of gamma delta T cells from tumor-infiltrating lymphocytes in humans [80].

Since molecules expressed on the surface of tumoral cells seem to be seen as an attractive criterion to draw the attention of γδ T cells, apolipoprotein A-1 (APOA-1) should not be left out of this discussion. A tumor recognition study led by Scotet’s group has shown that when in the presence of APOA-1, tumors expressing F1-ATPase can trigger the activation of Vγ9Vδ2 T cells by selectively binding to the Vγ9Vδ2 TCR [65].

### 3.8. Peptides

Despite the notoriety of non-peptidic antigens, there is a variety of protein-derived molecules that can trigger γδ T cell activation. In humans, the earliest evidence points to the tetanus toxoid of *Clostridium tetani*, capable of stimulating specific γδ T cell responses [47]. Another study has shown that processed Igλ light chains in humans [81], and even processed insulin in mice [82], are also recognized by γδ T cells. Additionally, cell surface-expressed proteinaceous ligands have been identified, including the classical MHC I and II molecules, as well as the non-classical MHC I-related molecule, T22, in mice [70], CD1c in humans [73], and CD1d in both mice and humans [72], as discussed above.

### 3.9. Aminoacyl-tRNA Synthetases (AA-RSs) 

Another study that aimed to investigate the antigen specificity of γδ-TCRs, in humans that develop myositis, could demonstrate that Vγ1.3Vδ2-TCR (M88) recognizes aminoacyl-tRNA synthetases (AA-RSs), an antigen that can also be targeted by an autoantibody called anti-Jo-1. The observation that AA-RSs are targeted by a γδ T cell and by an autoantibody reveals an unprecedented link between T cell and antibody responses in autoimmune myositis [83].

### 3.10. Dectin-1 (CLEC7A)

C-type lectin domain family 7 member A, also called dectin-1, is a human protein encoded by the CLEC7A gene that was initially identified by subtractive cDNA cloning, using mRNA extracted from murine DCs [84]. In addition to inducing Th17 cells, it was demonstrated that dectin-1 could also directly trigger the production of IL-17 by a subset of γδ T cells [85].

In 2014, it was demonstrated in a murine model of hepatic regeneration with the disruption of TCRδ and Clec7a, that γδT cells can regulate this process through the production of IL-22 and IL-17. This mechanism occurs by direct effects on hepatocytes and by the promotion of a regenerative phenotype in hepatic leukocytes. These observations suggest that dectin-1 is required for γδ T cell-promoted hepatic regeneration [86].

Table 1 summarizes the experimental pieces of evidence about γδTCR ligands, considering the differences between mouse and human γδ T cells.

### 3.11. IgG Antibodies as γδTCR Ligands

In almost all of the studies cited above, the search for a γδTCR ligand was focused on the identification of membrane-like or membrane-expressed molecules with no discussion about ligands that can have soluble forms, such as antibodies.

IgG antibodies are versatile molecules expressed by B cells and can also be detected in other cell membranes after interaction with Fcγ receptors. This molecule is also secreted by B cells, and its soluble form can reach almost all of the tissues of an organism.

Although, theoretically, these molecules may also interact specifically with γδTCRs, studies evaluating this hypothesis are very scarce in the literature. Among the few pieces of evidence in this issue, it was demonstrated that a monoclonal antibody that can recognize murine γδTCRs (clone UC7-13D5), induces the in vivo generation of γδ T cells [87]. Another study demonstrated that an anti-γδTCR antibody can be used to expand γδ T cells in vitro and may be used as cellular therapy in the treatment of lung cancer [88] or lymphoid malignancies [89]. These observations generate a very interesting fraction of new possibilities on each antibody that can act as a ligand of γδTCRs, and need to be discussed across a broader spectrum as will follow.

In 2017, it was hypothetically suggested that IgG could be a natural ligand of lymphocyte receptors, including γδTCRs, and mediate some functional modulations of γδ T cells according to an individual’s IgG repertoire. This hypothesis considered that this molecule could reach primary lymphoid organs, including the thymus, in soluble and membrane-expressed forms, and accurately interact with the clonal receptors expressed on those cells [90].

Very briefly, what underlies this hypothesis is the fact that mammalian organisms naturally have the potential to spontaneously produce IgG antibodies that express idiotypes capable of recognizing any natural protein [91]. This characteristic includes all self-proteins, hence all receptors are expressed in γδ T cells since their initial stages of maturation.

More recently, this hypothesis became experimentally evidenced with the demonstration that both murine and human IgG can modulate the frequency of IL-17-producing γδ T cells depending on the IgG donor’s immune status [92]. In the same study, it was demonstrated for the first time that murine IgG could directly interact with the thymic γδ T cell membrane (in the absence of FcγRs), although the co-localization of IgG and γδTCR has not been evaluated. Another recent study has also demonstrated that IgG can modulate the production of IFN-γ and IL-10 by human thymic γδ T according to the donor’s immune status [93]. Corroborating this hypothesis, similar studies evaluating the potential of IgG molecules as mediators of modulatory effects on lymphocytes during their maturation have demonstrated several effects on human TCD4, TCD8, iNKT, and B cells [94,95,96,97,98].

A direct relation between γδ T cell activation and the murine antibody-mediated immune response in an antigen-specific manner was also recently revealed [99]. In this study, the possible role of IgG as a γδTCR ligand was not evaluated, but the relationship between antigen-specific antibodies and γδ T cell activation in two murine models was shown [99]. This last piece of evidence demonstrated the importance of γδ T cells in protein-induced immune responses and autoimmunity.

So far, these observations cannot adequately explain the IgG role as a γδTCR ligand, but they could have been considered within the list of candidates for ligands of these receptors, as recently revised [39].

Considering that under in vivo conditions, IgG can reach thymic lymphocytes during its maturation process [100,101,102,103], these recent studies open a broad discussion, suggesting that IgG can exert a role modulating the maturation of γδ T cells at primary sites. Furthermore, the functional modulation mediated by IgG may vary according to individual repertoire specificity; it is possible that the role of γδ T cells in inducing tissue inflammation is influenced by the IgG response to antigen exposition and its natural IgG repertoire.

Very recently, a hypothesis called the “hooks without bait” theory was presented in the literature to discuss the role of natural and induced IgG repertoires as modulatory ligands, not only for γδ T cells, but in a broader context that includes T and B cells [104].

These observations are important in humans because of their wide variation in ambient exposure conditions and natural antibody repertoires, compared to murine observations obtained under controlled ambient exposure conditions and, therefore, homogeneous antibody diversity.

If the repertoire of IgG (the individual’s group of idiotypes) can influence γδ T cell functions by interacting with γδTCRs, they will modulate γδ T cells according to the expressed γ and δ chains. As discussed above, the expression of γ and δ chains is related to homing, functionality, and the antigen-specific recognition properties of γδ T cells. Therefore, if the IgG repertoire could indeed specifically interact with γδTCRs, this could result in the functional modulation of γδ T cells according to their antigen recognition pattern.

If confirmed, this hypothesis will corroborate the elucidation of several aspects of γδ T cell biology.

These biological aspects include the functional roles of γδ T cells in inflammation induced by different antigens [105], the γδTCR repertoire changes in response to infections [106], the constant γδTCR triggering of γδ T cells in vivo [107], the relationship between intestinal microbiota and γδ T cell functions [108], and the observation that the repertoire of intraepithelial γδ T cells is not biased toward thymic antigens [109].

**Figure 2:**
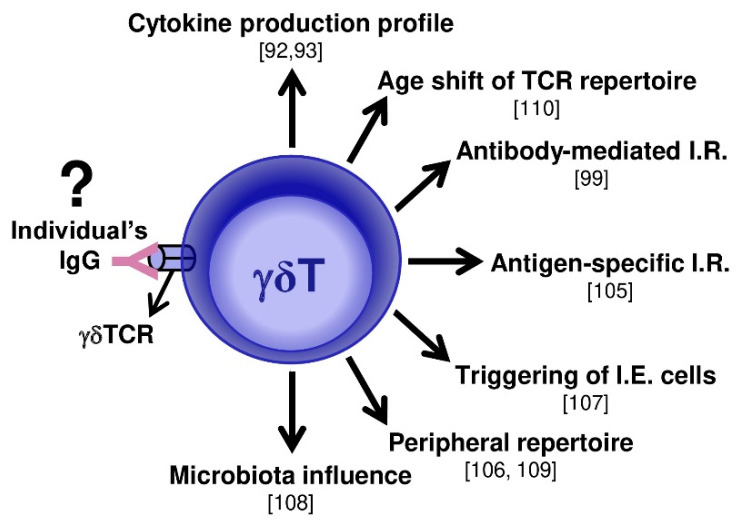
Possible γδ T cell-mediated mechanisms that can be influenced by IgG. The main immunologic mechanisms mediated by γδ T cells described in the literature, where IgG can be an influence by specifically interacting with γδTCRs, are illustrated with their respective references in brackets. ? = proposed non-evidenced ligand; I.R. = immune response; I.E. = intraepithelial.

A recent study demonstrated an additional γδ T cell characteristic that can be influenced by an individual’s IgG repertoire. It was described that an γδTCR’s repertoire shifts the Vγ- and Vδ-usage upon aging [110]. Considering that an individual’s IgG is certainly permissive to repertoire shifts with aging if IgG can indeed act as a ligand of γδTCRs, IgG will influence the frequency of γ and δ chain expression and modulate γδ T cell-mediated mechanisms, as suggested in Figure 2.

## 4. Concluding Remarks

Here we discuss one of the most intriguing unanswered questions of the immune system; indeed, the long period of searching for an efficient response reflects the high degree of difficulty in identifying the natural ligands of γδTCRs. Despite the problem, γδ T cells already represent a versatile immunoregulatory tool, since they can also be activated through cytokines without TCR engagement, allowing a faster response compared to αβ T cells. Moreover, they were able to kill target cells via death receptor-mediated apoptosis or the release of cytolytic granules. In this discussion, we seek a broader and philosophical approach about possible γδTCR ligands, not restricted to the physicochemical characteristics of molecular interactions, but considering the complexity of knowledge about the operating range of the immune system. The lack of biological evidence represents the primary limitation on our suggestion about the potential of IgG to answer this “immunological mystery”, but if confirmed, IgG molecules acting as a specific ligand of γδTCRs can influence several immune mechanisms.

Based on these observations, we can speculate that all the in vivo immunological mechanisms that involve γδ T cells are subject to influence by the IgG repertoire.

At this point, and maybe still for some years, we cannot be sure that each IgG repertoire has more affinity toward γδTCRs. However, considering some breakthrough works in the literature demonstrating the differential effects of IgG repertoires on γδ T, αβ T, and B cells, we can observe a common characteristic that yields some additional speculation about this mechanism. The main described functional effects of IgG repertoires were obtained from chronic pathophysiological conditions including allergies [92,93,95,96], atopic dermatitis [94,97], or HIV-1-infected patients [111]. Considering those observations, we suggest that the γδTCR/IgG affinity determination results form a complex, long-term, and not yet clarified, process.

If confirmed, these can be additional reasons for why such controversial observations about γδ T cell functions in the development of several diseases can be observed in the literature. Additionally, the verification of IgG as a ligand of γδTCRs will probably bring this molecule to a position of evidence in studies that aim to investigate immunoregulatory mechanisms involving γδ T cells, but under a new perspective where its recognition diversity starts to be considered.

Thus, we hope that the discussed aspects of γδ T cell biology can contribute to the future development of advances in antibody-based immunotherapies and vaccines.

## Figures and Tables

**Table 1:** γδTCR ligands with biological effects described in the literature. Classes of ligands and their effects on humans and mice.

Proposed Ligand	Class	Effect in Human γδ T Cells	Effect in Mouse γδ T Cells	References
Aminoacyl-tRNA synthetases (AA-RSs)	Enzyme	Destruction of skeletal muscle fibers in myositis.	Not reported in the literature due to the incapability ofgenerating transgenic mice.	[83]
Algal phycoerythrin (PE)	Protein	Production of IL-17.	Production of IL-17.	[66]
Annexin A2	Protein	Proliferation of a Vδ2^neg^ γδ T-cell subset.	Not reported in the literature.	[67]
Butyrophilin-3 (BTN3A)	Peptide	Intracellular PAg accumulation leading to activation of Vγ9Vδ2 T cells.	Not reported in the literature.	[68,69]
CD1c	MHC molecule	Lysis of CD1c-expressing tumor cells.	Not reported in the literature.	[71,72]
Cyanine 3 (Cy3)	Hapten	Not reported in the literature.	Up-regulation of CD44 in Cy3-specific γδ T cells, IL-17 production, and expression of receptors for IL-1 and IL-23.	[74]
4-hydroxy-3-nitrophenylacetyl (NP)	Hapten	No reported in the literature.	Up-regulation of CD44hi and CD62Llo (activated phenotype).	[74]
Mycobacterial antigen from *M. tuberculosis*	Non-peptide	Activates the Vγ2/Vδ2+.	In vivo activation of γδT and participation in the primary immune response to *M. tuberculosis*.	[75]
Monoethyl phosphate (MEP)	Non-peptide	Activates the Vγ2/Vδ2+.	Not reported in the literature since Vγ2Vδ2 T cells are restricted to primates.	[76]
TC22	MHC molecule	Not reported in the literature.	G8 bound T22 almost exclusively through its CDR3δ loop with only minor contacts from other CDR loops.	[70]
MICA/MICB	MHC class I-related molecules	Increase in the number of γδ T cells found in the tumoral tissue.	Not reported in the literature.	[77,78,79]
ULBP4	MHC class-related molecules	Expansion of γδ T cells from tumor-infiltrating lymphocytes.	Not reported in the literature.	[80]
Apolipoprotein A-I (APOA-I)	Protein	Activation of Vγ9Vδ2 T cells by tumors expressing F1-ATPase in the presence of APOA-1.	Not reported in the literature.	[65]
Dectin-1(CLEC7A)	Protein		Triggers the production of IL-17 by a subset of γδ T cells.	[86]
